# Childhood Vitamin A Capsule Supplementation Coverage in Nigeria: A Multilevel Analysis of Geographic and Socioeconomic Inequities

**DOI:** 10.1100/tsw.2010.188

**Published:** 2010-10-01

**Authors:** Olatunde Aremu, Stephen Lawoko, Koustuv Dalal

**Affiliations:** ^1^ Department of Public Health Sciences, Division of Social Medicine, Karolinska Institutet, Stockholm, Sweden; ^2^ College of Medicine, University of Ibadan, Oyo State, Nigeria; ^3^ Department of Medical and Health Sciences, Center for Medical Technology Assessment, Linkopings Universitet, Linkoping, Sweden

**Keywords:** childhood, community, coverage, geographic variation, inequity, multilevel analysis, Nigeria, neighborhood socioeconomic disadvantage, pediatric blindness, preschool, socioeconomic, supplementation, vitamin A capsule, vitamin A deficiency

## Abstract

Vitamin A deficiency (VAD) is a huge public health burden among preschool-aged children in sub-Saharan Africa, and is associated with a high level of susceptibility to infectious diseases and pediatric blindness. We examined the Nigerian national vitamin A capsule (VAC) supplementation program, a short-term cost-effective intervention for prevention of VAD-associated morbidity for equity in terms of socioeconomic and geographic coverage. Using the most current, nationally representative data from the 2008 Nigerian Demographic and Health Survey, we applied multilevel regression analysis on 19,555 children nested within 888 communities across the six regions of Nigeria. The results indicate that there was variability in uptake of VAC supplement among the children, which could be attributed to several characteristics at individual, household, and community levels. Individual-level characteristics, such as maternal occupation, were shown to be associated with receipt of VAC supplement. The results also reveal that household wealth status is the only household-level characteristic that is significantly associated with receipt of VAC, while neighborhood socioeconomic disadvantage and geographic location were the community-level characteristics that determined receipt of VAC. The findings from this study have shown that both individual and contextual socioeconomic status, together with geographic location, is important for uptake of VAC. These findings underscore the need to accord the VAC supplementation program the much needed priority with focus on characteristics of neighborhoods (communities), in addition to individual-level characteristics.

## INTRODUCTION

Vitamin A deficiency (VAD), which is characterized by visual impairment and lifetime blindness, remains a global health problem, afflicting more than 190 million preschool-aged children around the world[1], the majority of whom resides in developing countries[2]. In sub-Saharan Africa, VAD continues to be a major contributing factor to the increasing trends observed in childhood morbidity and mortality from infectious diseases in addition to pediatric blindness[2,3,4]. Periodic delivery of high-dose supplements have been established as the main strategy for combating VAD[5]. Effective coverage with a twice-yearly dose of vitamin A supplement delivered as a capsule is one such strategy. Vitamin A capsule (VAC) supplementation has proven to be a highly cost-effective child-survival intervention[6,7,8,9,10,11,12] and would be instrumental to achieving the Millennium Development Goal (MDGs) 4.

In Nigeria, VAD has been well documented as a long-time public health burden among preschool-aged children[13,14,15,16,17,18,19], with a high level of clustering across its regions[20]. Despite the morbidity and long-term consequences associated with VAD, and the successful integration of VAC supplementation into routine immunization exercises[21], less is known about the coverage of the VAC supplementation program among preschool-aged children in Nigeria. Understanding the pattern of VAC supplementation program coverage is important for improving the health outcome of children and their survival. Although numerous studies in sub-Saharan Africa[22,23,24] and globally[25,26,27,28] have examined and documented disparities in coverage of VAC supplementation based on individual characteristics, the influence of neighborhoods and the socioeconomic characteristics on coverage of VAC supplements has received less attention, whereas contextual characteristics of neighborhoods where these children reside may in themselves constitute a major disadvantage. For instance, it has been shown that residents of socioeconomically disadvantaged communities tend to have a higher mortality risk regardless of their individual demographic and socioeconomic characteristics[29,30]. In addition, the nexus between social and geographic stratification means that geographic location is another form of inequity as people from a lower socioeconomic status are often confined to the disadvantaged areas within the same country[31,32]. Thus, examining geographic coverage of population-based child-survival interventions becomes an essential part of preventive program monitoring.

To address limitations inherent with past studies on VAC supplementation coverage, and to further document the significance effect of contextual factors in population health programs, this study takes a step further, utilizing a multilevel logistic regression modeling approach[33], and examines inequities associated with VAC supplementation coverage among preschool-aged children in Nigeria. Multilevel logistic regression modeling is a technically robust statistical approach and has become more popular in the analysis involving clustered survey data that are often structured hierarchically. So, therefore, the main aim of this study was to examine how individual socioeconomic and geographic differentials, together with living in socioeconomically disadvantaged neighborhoods, determines VAC supplementation uptake among preschool-aged children in Nigeria. Quantifying inequities associated with coverage of VAC with a focus on proximate and contextual socioeconomic factors, in addition to geographic differentials, would serve as a means to improve the effectiveness of child-survival interventions in Nigeria.

## METHODS

### Data Source and Study Population

The dataset for this analysis was the 2008 Nigeria Demographic and Health Surveys (NDHS)[34], conducted between June and October 2008 in all six regions, which are comprised of 36 states and the Federal Capital Territory (FCT), Abuja, and was available as of December 2009. The NDHS was implemented by the National Population Commission (NPC) with technical assistance from ICF MEASURE DHS[35], and was funded by the U.S. Agency for International Development (USAID). The survey, which is a probability sample, is representative both at the national and residential levels. A

multistage stratified cluster sampling procedure was employed for sample collection. In the first stage, probability proportional to the size was used to select primary sampling units (PSUs); the size represents the number of households within the PSU. The PSUs are administratively defined areas and are usually used as proxies for communities or neighborhoods[36]. These terms, communities and neighborhoods, are used interchangeably in this study. The second stage involved a systematic sampling of households from each of the selected PSUs. Face-to-face interviews using a semi-structured questionnaire were then conducted in order to obtain information on various demographic and health characteristics from the respondents in the selected households. The questionnaire was translated into major languages and included information on children born to the respondents 5 years prior to the survey period and that of the household. The full details of the methods and procedure used in data collection in NDHS surveys has been published elsewhere[34].

Briefly, the overall goal of the NDHS surveys was to provide policy makers and health planners with information on various biodemographic, socioeconomic, and population health data from nationally representative samples of children aged 0–59 months, women aged 15–49 years, and men aged 15–59 years, so as to aid in the proper monitoring of population-based health interventions and future planning. The study populations for this analysis were 19,555 preschool-aged children within the age range of 12–59 months, nested within 888 communities across the six regions of Nigeria, and the members of their households who were participants for the 2008 NDHS.

## Ethical Considerations

This study is based on secondary analysis of existing survey data with all information that could be used to identify the respondents being removed. The field interviewers for the survey obtained informed consent from the mothers of the children included in this study and all questions were asked in close confidentiality. In addition, the survey instrument received ethical permission from the National Ethics Committee in the Federal Ministry of Health, Abuja and the institutional review board of ORC Macro Inc. The permission to use these data was obtained from Measure DHS and the Nigerian NPC[34].

## Measures

- **Outcome variables —** The main outcome variable in this study is the likelihood of a child 12 months of age and older having received a VAC supplement at least 6 months prior to the survey. This was assessed by the interviewer based on the report on the VAC supplementation card as shown by the mother who happens to be the respondent.
- **Explanatory variables —** Consistent with our research objective and given the effect of our outcome variable at more than one level where children are nested within households, which are in turn nested within communities, we considered two levels of explanatory variables[37]. Level 1 contained individual characteristics (of both child and parents) and that of the community at level 2, which included the characteristics of the communities in which each child resided.
- **Individual-level explanatory variables —** These are various children's and household-level characteristics, and are described in Table 1.
- **Community-level explanatory variables —** Communities were defined basically through grouping of clusters otherwise known as PSUs and were used as proxy for neighborhood. Places of residence were grouped based on location as rural or urban. Geographic regions were characterized as (1) North Central, (2) North East, (3) North West, (4) South East, (5) South South, and (6) South West. The neighborhood socioeconomic disadvantage index was developed using principal component analysis (PCA)[38]. This comprised four variables: proportion of respondents living in rural areas, proportion of respondents who were unemployed, proportion of respondents living below the poverty level (below the 20% quintile), and proportion of respondents

**TABLE 1**
**Definitions and Measure of Individual-Level Variables Used in the Study**

| Variables | Measures |
|---|---|
| Child's sex | Grouped as male or female |
| Child's age (months) | Grouped as 12–23, 24–35, 36–47, 47+ months |
| Mother's age (years) | Grouped as 15–24, 25–34, 35+ years |
| Mother's education | Grouped as no education, primary, secondary, and higher |
| Father's education | Grouped as no education, primary, secondary, and higher |
| Mother's occupation | Grouped as not working, manual, professional |
| Father's occupation | Grouped as not working, manual, professional |
| Household wealth index | Household wealth index was constructed based on possession of durable items, such as radio set, refrigerator, television, and motorcar; quality of dwelling, such as floor type or roof type using PCA. This resulting index was then grouped as poorest, poorer, middle, rich, richest. |

who are uneducated. This methodology has been used by many others studying the effect of neighborhood socioeconomic status on health[39,40]. The scores generated from the continuous index have a mean value of 0 and standard deviation of 1, and were used to classified neighborhoods into two categories: (1) most disadvantaged, if the scores are higher, which implies a lower socioeconomic position; and (2) least disadvantaged for the lower scores, in which case is higher socioeconomic position.

## Analytical Procedures

This study employed multilevel logistic modeling techniques[37,41,42,43] in order to account for the hierarchical structure of the DHS data and the binary response of our outcome variable. A multilevel logistic analysis permits the inclusion of explanatory variables at more than one level of hierarchy, and further allows for the clustering effect of the outcome variable at both the individual and community level to be taken into consideration[33,44,45,46,47].

### *Multilevel Logistic Regression Modeling*

We applied a three-level multilevel logistic model to the data sample with focus on the effect of different explanatory variables on the binary outcome variable. The model is written thus

$$\log it\left(\pi_{ijk}\right) = \log\left(\frac{\pi_{ijk}}{1-\pi_{ijk}}\right) = \beta_0 + \boldsymbol{X}_{ijk} + \boldsymbol{u}_{0jk} + \boldsymbol{v}_{0k} \tag{1}$$

Where $\pi_{ijk}$ is the probability of a child $i$ residing in community $j$ in region $k$ having received a dose of VAC. The right-hand side of the equation consists of fixed parts $X_{ijk}$ and $\beta_0$, which are basically estimating the vectors attributable to the explanatory coefficients at both individual and community levels, respectively, and the last two vectors, $u_{0jk}$ and $v_{0k}$, are the random effects that denote unobserved factors at both the child and community level, respectively.

### *Model Specification*

We specified a three-level model as follows: Model 1, the empty model, has no explanatory variable included; Model 2 (individual characteristic model) controls for a set of individual-level explanatory variables at the level of the child and households; Model 3 (community model) systematically adjusts for both individual- and community-level variables.

### *Fixed Effects*

This estimates the association between the likelihood of receiving VAC supplement and various explanatory variables expressed as odds ratio (OR) at their 95% confidence intervals (95% CIs).

### *Random Effects*

This is otherwise known as “measures of variation” and was expressed as variance partition coefficient (VPC) and proportional change in variance (PCV)[48]. The VPC measures the likelihood of receipt of VAC supplement that is attributable to community level. A large VPC value indicates a high clustering of receipt of VAC supplement in the communities, and a low VPC value reflects homogeneous likelihood of receipt of VAC supplement. All analyses were performed using the logit link function in STATA 11.0 for windows (Stata Corp, Inc., TX). All parameters were estimated using adaptive Gaussian quadrature (AGQ) maximum likelihood estimator[49], with $p < 0.05$ considered statistically significant. We used Deviance Information Criterion (DIC) estimates to appraise the fitness of our model, with successive decrease in the value of DIC indicating a well-fitted model.

## RESULTS

### Descriptive Statistics

The individual and contextual characteristics of the study sample by VAC supplementation status are as shown in Table 2. In all, a total of 19,555 children at level 1, nested within 888 communities at level 2, were used in the analysis. Only 3,887 representing 19.8% had received VAC supplement and, of these, almost 38% were between the age of 12 and 23 months. There were slight differences in VAC supplement uptake rate between male and female children, 20.1 and 19.7%, respectively. The majority of children who received VAC supplement was likely to be born to mothers belonging to the highest strata of the wealth index, manual workers with no education, and those who live in urban area within low socioeconomically disadvantaged neighborhoods.

### Multilevel Models

Table 3 depicts multilevel logistic regression results for log odds of receipt of VAC supplement. The empty model shows that there is a significant variability in the log odds of receiving VAC supplement across the communities ($\tau = 1.41$, $p = 0.001$). Based on the intracommunity correlation coefficients estimated by intercept variance component, 30% of variability in the log odds of a child receiving VAC supplement is due to community-level factors. Model 2 adjusted for the following explanatory variables: age of the child, sex, mother’s occupational status and educational attainment, father’s occupation and educational attainment, and household wealth status. This adjustment shows that log odds of receiving VAC supplement was 20% lower (OR = 0.80; 95% CI 0.71, 0.89) for children of ages 24–35 months and

**TABLE 2**
**Sample Characteristics of Proportion of Children, Aged 12–59 Months, by VAC Supplementation Status Based on NDHS 2008**

| Variables | Received VAC | | |
|---|---|---|---|
| | Yes | No | |
| | N (%) | N (%) | Total N (%) |
| Child's age (months) | | | |
| 12–23 | 1,477 (17.3) | 7,048 (82.7) | 8,525 (43.6) |
| 24–35 | 839 (23.1) | 2,790 (76.9) | 3,629 (18.6) |
| 36–47 | 839 (21.6) | 3,053 (78.4) | 3,892 (19.9) |
| 48+ | 732 (20.9) | 2,777 (79.1) | 3,509 (17.9) |
| Child's sex | | | |
| Male | 1,985 (20.1) | 7,914 (79.9) | 9,899 (50.6) |
| Female | 1,902 (19.7) | 7,754 (80.3) | 9,656 (49.4) |
| Mother's age (years) | | | |
| 15–24 | 784 (15.5) | 4,260 (84.5) | 5,044 (25.8) |
| 25–34 | 2,036 (21.5) | 7,446 (78.5) | 9,482 (48.5) |
| 35+ | 1,067 (21.2) | 3,962 (78.8) | 5,029 (25.7) |
| Mother's education | | | |
| No education | 230 (44.2) | 291 (55.8) | 521 (2.7) |
| Primary | 1,679(14.2) | 10,163 (85.8) | 11,842 (60.6) |
| Secondary and higher | 1,978(27.5) | 5,214 (72.5) | 7,192 (36.7) |
| Father's education | | | |
| No education | 583 (32.7) | 1,199 (67.3) | 1,782 (9.1) |
| Primary | 1,316 (13.8) | 8,196 (86.2) | 9,512 (48.6) |
| Secondary and higher | 1,988 (24.1) | 6,273 (75.9) | 8,261 (42.3) |
| Mother's occupation | | | |
| Not working | 1,025 (15.0) | 5,783 (85.0) | 6,808 (34.8) |
| Manual | 2,301 (22.7) | 7,825 (77.3) | 10,126 (51.8) |
| Professional | 543 (21.6) | 1,968 (78.4) | 2,511 (12.8) |
| Missing + | 18 (16.4) | 92 (83.6) | 110 (0.6) |
| Father's occupation | | | |
| Not working | 432 (30.5) | 984 (69.5) | 1,416 (7.2) |
| Manual | 2,388 (17.5) | 11,250 (82.5) | 13,638 (69.7) |
| Professional | 1,032 (24.1) | 3,249 (75.9) | 4,281 (21.9) |
| Missing + | 35 (15.9) | 185 (84.1) | 220 (1.2) |
| Wealth index | | | |
| Poorest | 791 (12.9) | 5,352 (87.1) | 6,143 (31.4) |
| Poorer | 866 (16.3) | 4,458 (83.7) | 5,324 (27.2) |
| Middle | 881 (23.1) | 2,931 (76.9) | 3,812 (19.5) |
| Richer | 731 (27.5) | 1,927 (72.5) | 2,658 (13.6) |
| Richest | 618 (38.2) | 1,000 (61.8) | 1,618 (8.3) |

*Table 2 continues*

**TABLE 2 (continued)**

| **Community-Level Variables** | **Received VAC** | | |
|---|---|---|---|
| | **Yes** | **No** | |
| | **N (%)** | **N (%)** | **Total N (%)** |
| Neighborhood economic disadvantage[a] | | | |
| Low | 2,436 (62.7) | 7,398 (47.2) | 9,834 (50.3) |
| High | 1,451 (37.3) | 8,270 (52.8) | 9,721 (49.7) |
| Place of residence | | | |
| Rural | 2,719 (17.9) | 12,504 (82.1) | 15,223 (77.8) |
| Urban | 1,168 (27.0) | 3,164 (73.0) | 4,332 (22.2) |
| Geographic region | | | |
| North Central | 898 (23.4) | 2,947 (76.6) | 3,845 (19.7) |
| North East | 953 (17.8) | 4,414 (82.2) | 5,367 (27.4) |
| North West | 843 (13.1) | 5,597 (86.9) | 6,440 (32.9) |
| South East | 139 (17.3) | 665 (82.7) | 804 (4.1) |
| South West | 422 (28.5) | 1,057 (71.5) | 1,479 (7.6) |
| South South | 632 (39.0) | 988 (61.0) | 1,620 (8.3) |

**+** Missing values were excluded from the final analysis, values might not add up to 100%.

[a] For analysis, the principal component of neighborhood socioeconomic disadvantage means (SD) was used. Data source: 2008 Nigeria Demographic and Health Survey**.**

17% higher (OR = 1.17; 95% CI 1.02, 1.33) for children of ages 36–47 months, compared with the reference category (12–23 months). Children born to mothers aged 15–24 years had a 20% lower log odds of receiving VAC supplement (OR = 0.80; 95% CI 0.70, 0.90) as compared with children of mothers aged 35 years and above the reference category. Surprisingly, children born to mothers with at least primary, secondary, or higher education had 56% (OR = 0.44; 95% CI 0.34, 0.60) and 31% (OR = 0.69; 95% CI 0.54, 0.89) lower log odds of receiving VAC supplement as compared to the children whose mothers were not educated at all (reference group). Likewise, children born to fathers with primary education had a 23% (OR = 0.77; 95% CI 0.64, 0.92) lower log odds of receiving VAC supplement compared to their peers whose fathers were not educated. In line with expectation, compared to the children whose mothers were not working, the children whose mothers are in the professional and manual working categories had 29% (OR = 1.29; 95% CI 1.12, 1.50) and 34% (OR = 1.34; 95% CI 1.20, 1.50) significantly higher log odds of receiving VAC supplement, respectively, whereas, the log odds of receiving VAC supplement was 26% (OR = 0.84; 95% CI 0.71, 0.99) lower for children whose fathers were manual workers as compared to those whose fathers were not working at all.

However, compared to children from the poorest households, children from the wealthiest households had a significantly more than double log odds of receiving VAC supplement (OR = 2.37; 95% CI 1.88, 2.90); in addition, children from the households in the fourth quintile of the wealth index had a 82% (OR = 1.82; 95% CI 1.51, 2.20) higher log odds of receiving VAC supplement as compared to their counterparts from the poorest households. Also, compared to the children from the poorest households, children from households at the middle-class of the wealth index and those from the least poor households had almost 60% (OR = 1.59; 95% CI 1.35, 1.86) and 19% (OR = 1.19; 95% CI 1.04, 1.37) higher log odds of receiving VAC supplement, respectively. This adjustment indicated that there is still significant variability in log odds of receiving VAC supplement ($\tau$ = 1.19, $p$ = 0.001), with slight decrease in community-level variance as compared to the empty model. As reported by the PCV, 16% of variability in log odds of receiving VAC supplement across communities was explained by various individual child

**TABLE 3**
**Multilevel Logistic Regression Modeling of Factors Associated with VAC Supplement Uptake Among Nigerian children, NDHS 2008**

| Variables | Model 1 [a] | Model 2 [b] | Model 3 [C] |
|---|---|---|---|
| | OR (95% CI) | OR (95% CI) | OR (95% CI) |
| **Fixed effects** | | | |
| Child's age in months | | | |
| 12–23 (ref) | | 1.00 | 1.00 |
| 24–35 | | 0.80 (0.71–0.89)*** | 0.80 (0.71–089)*** |
| 36–47 | | 1.17 (1.02–1.33)** | 1.17 (1.02–1.33)** |
| 48+ | | 1.06 (0.97–1.25) | 1.09 (0.96–1.24) |
| Child's sex | | | |
| Male (ref) | | 1.00 | 1.00 |
| Female | | 0.96 (0.89–1.05) | 0.96 (0.89–1.05) |
| Mother's age (years) | | | |
| 35+ (ref) | | 1.00 | 1.00 |
| 15–24 | | 0.80 (0.70–0.90)** | 0.81 (0.72–0.92)** |
| 25–34 | | 0.92 (0.84–1.02) | 0.93 (0.84–1.03) |
| Mother's education | | | |
| No education (ref) | | 1.00 | 1.00 |
| Primary | | 0.44 (0.34–0.60)*** | 0.47 (0.36–0.61)*** |
| Secondary and higher | | 0.69 (0.54–0.89)*** | 0.70 (0.55–0.89)** |
| Father's education | | | |
| No education (ref) | | 1.00 | 1.00 |
| Primary | | 0.77 (0.64–0.92)** | 0.72 (0.65–0.92)** |
| Secondary and higher | | 0.90 (0.77–1.06) | 0.87 (0.74–1.03) |
| Mother's occupation | | | |
| Not working (ref) | | 1.00 | 1.00 |
| Manual | | 1.34 (1.20–1.50)*** | 1.30 (1.17–1.44)*** |
| Professional | | 1.29 (1.12–1.50)*** | 1.27 (1.10–1.47)** |
| Father's occupation | | | |
| Not working (ref) | | 1.00 | 1.00 |
| Manual | | 0.84 (0.71–0.99)* | 0.84 (0.71–1.00)* |
| Professional | | 0.93 (0.78–1.11) | 0.91 (0.76–1.09) |
| Wealth index | | | |
| Poorest(ref) | | 1.00 | 1.00 |
| Poorer | | 1.19 (1.04–1.37)* | 1.11 (0.96–1.27) |
| Middle | | 1.59 (1.35–1.86)*** | 1.37 (1.16–1.62)*** |
| Richer | | 1.82 (1.51–2.20)*** | 1.45 (1.21–1.82)*** |
| Richest | | 2.37 (1.88–2.90)*** | 1.86 (1.45–2.38)*** |

*Table 3 continues*

**TABLE 3 (continued)**

| Community-Level Variables | Model 1 [a] | Model 2 [b] | Model 3 [C] |
|---|---|---|---|
| | OR (95% CI) | OR (95% CI) | OR (95% CI) |
| Geographic region | | | |
| North Central (ref) | | | 1.00 |
| North East | | | 0.87 (0.63–1.19) |
| North West | | | 0.45 (0.34–0.61)*** |
| South East | | | 0.58 (0.36–0.92)** |
| South West | | | 1.01 (0.70–1.47) |
| South South | | | 1.66 (1.17–2.35)*** |
| Residence | | | |
| Rural (ref) | | | 1.00 |
| Urban | | | 1.21 (0.87–1.67) |
| Community economic status | | | |
| Neighborhood socioeconomic disadvantage index | | | 0.76 (0.65–0.89)** |
| **Random effects** | | | |
| Intercept | 0.18 (0.16–0.21)*** | 0.30 (0.22–0.41)*** | 0.34 (0.21–0.53)*** |
| Community-level variance (SE) | 1.41 (0.05)*** | 1.19 (0.04)*** | 1.13 (0.04)*** |
| VPC (%) | 30 | 26.5 | 25.5 |
| Explained variation PCV (%) | Reference | 15.6 | 19.9 |
| **Model fit statistics** | | | |
| DIC (-2log likelihood) | 16,914 | 16,524 | 16,444 |

Abbreviations: OR, odds ratio; CI, confidence interval; SE, standard error; DIC, deviance information criterion ***p** < 0.05, ****p** < 0.01, and *****p** < 0.001; ref, reference category; VPC, variance partition coefficient; PCV, proportional change In variance

[a] Model 1 is null model with no exposure variable.

[b] Model 2 is adjusted for child's characteristics (age, sex), mothers and fathers characteristics (education, occupation, and wealth index).

[c] Model 3 sequentially adjusted for community socioeconomic disadvantage, place of residence, and region, in addition to all the included variables in Model 2.

and household-level compositional characteristics at the community level. Model 3, in Table 3, added the community-level variables that included region of residence, place of residence, and community-level socioeconomic disadvantage, in addition to variables in Model 2, to ascertain their effects on log odds of a child receiving VAC supplement. As can be seen from Table 3, the log odds of receiving VAC supplement by children born to mothers living in the North West and South East is lowered by 55% (OR = 0.45; 95% CI 0.34, 0.61) and 42% (OR = 0.58; 95% CI 0.36, 0.92), respectively, as compared to those living in North Central (reference category). Independent of other factors, children of mothers living in the South South region had 66% higher log odds of receiving VAC supplement compared to those of the reference category. For every one unit increase in standard deviation (SD) of community socioeconomic disadvantage index, the log odds of receiving VAC supplement decreased by 24% (OR = 76; 95% CI 0.65, 0.89). As shown by the intracommunity correlation coefficient; in comparison to Model 2, the community-level variability in log odds of receiving VAC supplement remains significant ($\tau$ = 1.13, $p$ = 0.001), with 20% of the variance being attributed to both individual- and community-level variables in Model 3. Thus, after controlling for various variables at both individual and contextual levels, the household wealth index and community level socioeconomic disadvantage index, along with region of residence, remains statistically significant.

## DISCUSSION

The result of this study shows that individual-, household-, and community-level socioeconomic characteristics, along with geographic location, were the major determinants of VAC supplement uptake among Nigerian children based on the 2008 NDHS. This study, to the best of our knowledge, is the first nationally representative population-based study to document the effect of socioeconomic development of neighborhoods on likelihood of receipt of VAC supplement using multilevel methodology. Our results show that children living in highly socioeconomically disadvantaged communities were 24% less likely than their peers residing in less socioeconomically disadvantaged communities to receive VAC supplements. This implies that children who were residents of high socioeconomically disadvantaged communities were likely to have mothers who were not working, are rural dwellers, and belong to the lowest 20% of the wealth quintile.

However, the effect of household wealth status, which had been demonstrated in previous studies[50,51] to be an important factor for availing the uptake of VAC supplements, was also confirmed by our study, showing that children from the richest households have a higher likelihood of receiving VAC supplement as compared to their peers from the poorest households. This finding, however, is in contrast to those reported by a study in Tanzania[52], where there were no differences in supplementation rate based on household wealth status.

Individual measures of maternal or paternal socioeconomic characteristics, such as education and occupation, had been shown to increase the uptake of some preventive child-survival intervention strategies, including vaccines[53] and vitamin A supplementation[25,50]. Our study, however, yielded mixed findings with regards to their roles in VAC supplementation among Nigerian preschool children. More specifically, our study showed that with all other factors controlled for children born to mothers with primary, secondary, or higher education compared to their peers whose mothers were not educated at all were less likely to receive VAC supplement. Similar findings were obtained for the role of father's education with the odds in favor of children whose fathers were not educated for likelihood of receiving VAC supplement. This contraintuitive finding is not new, especially with regards to uptake of childhood preventive intervention. A probable reason for our findings might be related to what has been reported earlier in some studies on childhood immunization coverage in Nigeria, where it was shown that children whose mothers had primary education are less likely to be immunized compared with those whose mothers were not educated[54]. It has also been reported that effect of maternal education on immunization uptake sometimes becomes nonsignificant when modeled together with paternal education, which might be the case in our analysis[54,55,56]. In addition, the fact that the VAC supplementation program is normally carried out sometimes along with the routine immunization program might have lent support for the findings in our study.

The findings that children whose mothers were professional workers or who were in manual occupations were more likely to receive VAC supplement as compared to those whose mothers were not working is in the right direction. This is, however, in contrast to a study in Bangladesh[51], where it was shown that children whose mothers were not involved in any income-generating activities were more likely to receive VAC supplement compared to those whose mothers were actively engaged in income-generating activity. Also, this present study indicates that the father's occupation is not in any way protective for receipt of VAC supplement, as children whose fathers were manual workers were less likely to receive VAC supplement compared to those whose fathers were not working. Furthermore, our results show that there is wide variation in uptake of VAC supplement by children across all the geographical regions in Nigeria; this finding is similar to those obtained in another western African country[57]. Specifically, the results show that despite the reported high prevalence of VAD, which is higher than the national average in the North West geographical region[19], children in this region are less likely to have received VAC supplement. Of particular concern in the North West geographic region, however, is the dry savannah and low level of annual rainfall, which means legumes and cereal are the principal crops widely available in the region. As a consequence, children and their families would consume less of the foods rich in vitamin A. The results also indicate that children living in the South East region, compared to those in the North Central region, were less likely to receive VAC supplement. In contrast to the disparities in receipt of VAC supplement observed for children from both North West and South East regions, children from the South South region were more likely to receive VAC supplements. Similarly, children from the South West region have equal likelihood of receiving VAC supplement as their counterparts from the North Central region. Unlike what has been documented elsewhere[28], our study found no association between place of residence (e.g., rural or urban) on likelihood of receipt of VAC supplement. Hence, our findings were in the same direction with what was reported among children in Tanzania[52].

## STUDY LIMITATIONS AND STRENGTH

The findings from this study are not without limitations and should be noted. First, this study used an indirect measure of household wealth status. The demographic and health survey does not collect information on personal income, as it is rather difficult to obtain such data in a developing country setting such as Nigeria. Hence, the use of an asset-based index as a proxy for household wealth has become the most widely used methodology and has been found to be reliable[58]. Second, other proximate and contextual-level determinants, such as social norms and beliefs that are known to be important for uptake of preventive child health intervention and were not included in the model, are another potential limitation. Third, our use of community as proxy for administratively defined boundaries might generate biases. Despite these limitations, our study has numerous strengths. First, the study is based on the most recent nationally representative population-based survey sample, which cuts across all the regions of the country. Second, the DHS surveys are similar in design, with similar variables that are comparable across settings; therefore, the findings could be generalized to other developing countries. Third, this study is unique in it use of the multilevel-modeling technique, which takes into consideration the nested structure of the data, thus allowing for the clustering effect of the outcome variable to be examined[45], which is an important fact that has been ignored by previous studies. Fourth, neighborhood-level socioeconomic characteristics have been shown to be more well correlated than individual-level measures of socioeconomic characteristics[59]; hence, our use of this approach in this study is justified.

## CONCLUSION AND POLICY IMPLICATION

This population-based multilevel analysis of the VAC supplementation program adds to our understanding of socioeconomic and geographic inequities associated with coverage of preventive childhood public health interventions in Nigeria. This study has further revealed, as is often the case for most preventive child health interventions, that children from wealthier households benefit more and are being reached by newer interventions, while those from poorer households continue to be left out[60]. In the light of the findings from this study, there is need for pragmatic efforts that should aim at improving the uptake of VAC supplement across all the regions, with all the children from all strata of the socioeconomic index benefiting. In particular, the federal government of Nigeria should, in conjunction with the state government across the regions and in partnership with donor agencies, adopt a continual scaling up of VAC supplementation to reduce inequity in coverage across all the agroecological zones of the country.

## ACKNOWLEDGMENT

The authors are grateful to Measure DHS for providing them with the DHS data and to Prof. Anders Hjern of the Centre for Health Equity Study for his thoughts during the preparation of the revised version of the paper. The authors also thank the two anonymous reviewers for their critical review of this manuscript, and finally, the authors wish to extend their gratitude to the handling editor Prof. Joav Merrick.
